# Generation of tooth-like structures from integration-free human urine induced pluripotent stem cells

**DOI:** 10.1186/2045-9769-2-6

**Published:** 2013-07-30

**Authors:** Jinglei Cai, Yanmei Zhang, Pengfei Liu, Shubin Chen, Xuan Wu, Yuhua Sun, Ang Li, Ke Huang, Rongping Luo, Lihui Wang, Ying Liu, Ting Zhou, Shicheng Wei, Guangjin Pan, Duanqing Pei

**Affiliations:** 1 CAS Key Laboratory of Regenerative Biology and Guangdong Provincial Key Laboratory of Stem Cell Biology and Regenerative Medicine, South China Institute for Stem Cell Biology and Regenerative Medicine, Guangzhou Institute of Biomedicine and Health, Chinese Academy of Sciences, 190 Kai Yuan Avenue, Science Park, Guangzhou, 510530 P.R.China; 2 Department of Regeneration Medicine, School of Pharmaceutical, Jilin University, Changchun, P.R. China; 3 Department of Oral and Maxillofacial Surgery, Laboratory of Interdisciplinary Studies, School and Hospital of Stomatology, Peking University, Beijing, P.R. China; 4 Biodynamic Optical Imaging Center (BIOPIC), Peking University, Beijing, P.R. China; 5 The Shenzhen Key Lab of Gene and Antibody Therapy, Center for Biotech & Biomedicine and Division of Life Sciences, Graduate School at Shenzhen, Tsinghua University, Shenzhen, P.R. China; 6 Center for Biomedical Materials and Tissue Engineering, Academy for Advanced Interdisciplinary Studies, Peking University, Beijing, P.R. China

**Keywords:** Human urine, Integration-free iPSCs, Recombinant tooth, Bioengineered tooth, Dental epithelium

## Abstract

**Background:**

Tooth is vital not only for a good smile, but also good health. Yet, we lose tooth regularly due to accidents or diseases. An ideal solution to this problem is to regenerate tooth with patients’ own cells. Here we describe the generation of tooth-like structures from integration-free human urine induced pluripotent stem cells (ifhU-iPSCs).

**Results:**

We first differentiated ifhU-iPSCs to epithelial sheets, which were then recombined with E14.5 mouse dental mesenchymes. Tooth-like structures were recovered from these recombinants in 3 weeks with success rate up to 30% for 8 different iPSC lines, comparable to H1 hESC. We further detected that ifhU-iPSC derived epithelial sheets differentiated into enamel-secreting ameloblasts in the tooth-like structures, possessing physical properties such as elastic modulus and hardness found in the regular human tooth.

**Conclusion:**

Our results demonstrate that ifhU-iPSCs can be used to regenerate patient specific dental tissues or even tooth for further drug screening or regenerative therapies.

**Electronic supplementary material:**

The online version of this article (doi:10.1186/2045-9769-2-6) contains supplementary material, which is available to authorized users.

## Background

The goal of regenerative medicine is to regenerate fully functional tissues or organs that can replace lost or damaged ones occurred during diseases, injury and aging [1, 2]. The advent of iPSCs should speed up the application of regenerative tissues or organs such as tooth in the clinic [3]. While iPSC-derived cells have been tested in animal models [4–7], no solid organs or tissues such as tooth have been generated with human iPSCs. The tooth represents one of the best experimental models in organogenesis [8, 9], and is easily accessible for human replacement therapy [10]. Tooth is formed by reciprocal interactions between epithelium and mesenchymal cells derived from the cranial neural crest [11, 12]. Developmentally, the odontogenic potential shifts from the dental epithelium to dental mesenchyme at bud stage (Embryonic day 12, E12) [13, 14]. Then, the epithelium differentiates into ameloblasts and finally forms the enamel, while the mesenchyme differentiates into the dentin, cementum and dental pulp [15]. Tooth stem cells such as dental pulp stem cells (DPSCs), periodontal ligament stem cells (PDLSCs), and gum stem cells (GSCs) have been isolated and investigated for tooth regeneration [16–18]. Recently, Arakaki and colleagues reported that mouse iPSCs (miPSCs) could be differentiated into ameloblasts via interactions with dental epithelium and these miPSCs derived epithelial cells were positive with the epithelial cell markers p63, cytokeratin-14 (K14), and ameloblast markers ameloblastin and enamelin [19]. Shortly after, another group differentiated miPSCs into neural crest-like cells to generate odontoblasts that express dental mesenchyme markers Msx1, Pax9, Lhx6, and odontoblast marker dentin sialoprotein (DSP) [20]. Recently, miPSCs were mixed with dissociated mouse dental epithelial and mesenchymal cells and found to contribute to odontogenesis *in vivo*[21]. However, human iPSCs have not yet been explored for tooth regeneration.

We developed a chimeric culture system for tooth regeneration from human iPSCs (Additional file 1). We have a collection of both H1 hESCs as a control and iPSCs derived in our laboratories, mostly from human urine cells (hU) by oriP/EBNA episomal vectors carrying a combination of reprogramming factors Oct4, Sox2, SV40LT, Klf4 and miR302/367 through electroporation [22, 23]. We hypothesized that hESCs or hiPSCs can be induced to produce epithelial sheets capable of replacing the E14.5 mouse dental epithelium in a reconstitution process (Additional file 1). The reconstituted explants can then be cultured *in vitro* for 1–2 days and transplanted beneath mouse subrenal capsule for 3 weeks for tooth regeneration (Additional file 1).

## Results

### Epithelial sheets generated from hESCs and iPSCs

**Figure 1:**
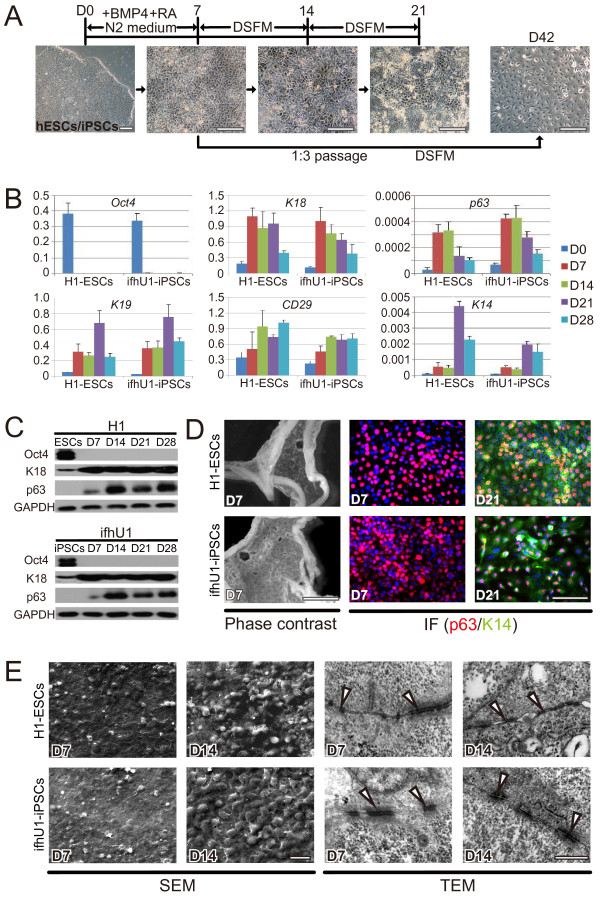
**hESC**/**iPSC derived epithelial lineages.** **(A)** Representative the epithelial differentiation process of hESCs or hiPSCs through supplement of RA and BMP4 in N2 medium for 7 days, then either changing into DSFM directly or passaging at a split ratio of 1:3 for culture continuously. Scale bar corresponds to 200 μm. **(B)** qPCR of representative experiment showing the down regulation of ESC-specific transcription factor (*Oct4*) and up regulation of keratinized epithelial markers (*K18*, *p63*, *K19*, *CD29*, *K14*). **(C)** Western blot for Oct4, p63, and K18 of lysates from H1 or ifhU1-iPSCs derived epithelial cells; GAPDH is the loading control. **(D)** Phase contrast captures and immunofluorescence staining [IF: p63 (red), K14 (green), DAPI (blue)] of epithelial sheets derived from H1 and ifhU1-iPSCs at D7 and D21. Scale bars correspond to 2000 and 200 μm, respectively. **(E)** Scanning electron microscope (SEM) and transmission electron microscopy (TEM) images of H1-ESCs and ifhU1-iPSCs derived epithelial sheets at D7 and D14. White arrowheads indicate desmosomes between epithelial cells of the sheet. Scale bars in SEM and TEM correspond to 20 and 0.5 μm respectively.

We first must devise a way to obtain dental epithelia from hESCs or ifhU-iPSCs and decided on a stage-specific approach based on retinoic acid (RA) and bone morphogenetic protein 4 (BMP4) in N2 medium [24]. To this end, we obtained epithelial cells with keratinocyte-like morphology at D7 (Figure 1A). When passaged with a ratio at 1:3, these cells became definitive keratinocytes in a defined keratinocyte serum-free medium (DSFM) at D42 (Figure 1A). However, these cells survive poorly after passage. We then allowed the cells to grow as epithelial sheets without passage, and they became denser at D14, and detached slightly with some cell death at D21 (Figure 1A). The differentiating cells at D7, 14, 21, 28 were harvested and investigated for the expression of pluripotent and keratinocyte progenitor’s markers by qPCR and Western blot. During epithelial differentiation, H1-ESCs and ifhU-iPSCs behaved similarly as shown with markers examined at RNA level (*Oct4*, *K18*, *p63*, *K19*, *CD29*, and *K14*), showing up regulated expressions in epithelial markers and down-regulation of pluripotent marker *Oct4* (Figure 1B). Similar trends were also observed for protein expression (Oct4, K18 and p63, Figure 1C). Moreover, the expression of p63 and K14 were verified by immunofluorescence. p63 was detected earlier at D7 and continuously expressed at D21, while K14 expression was detected later at D21 (Figure 1D). As a result, we obtained homogenous layers of epithelial cells as sheets from both hESCs and ifhU-iPSCs at D7 (Figure 1D). These sheets harvested at D7 were tenacious and flexible, showing the flat and smooth surface at the apical side as observed by scanning electronic microscopy (SEM) (Figure 1E). The sheets became rugged with prominent nuclei at D14 (Figure 1E). Under transmission electron microscopy (TEM), the desmosomes could be observed clearly between the epithelial cells at both D7 and D14 (Figure 1E). Together, these results suggest that the epithelial sheets generated at D7 from hESCs or ifhU-iPSCs have desired properties for being induced for tooth regeneration.

### Tooth-like structures generated from hESC and iPSC derived epithelial sheets

**Figure 2:**
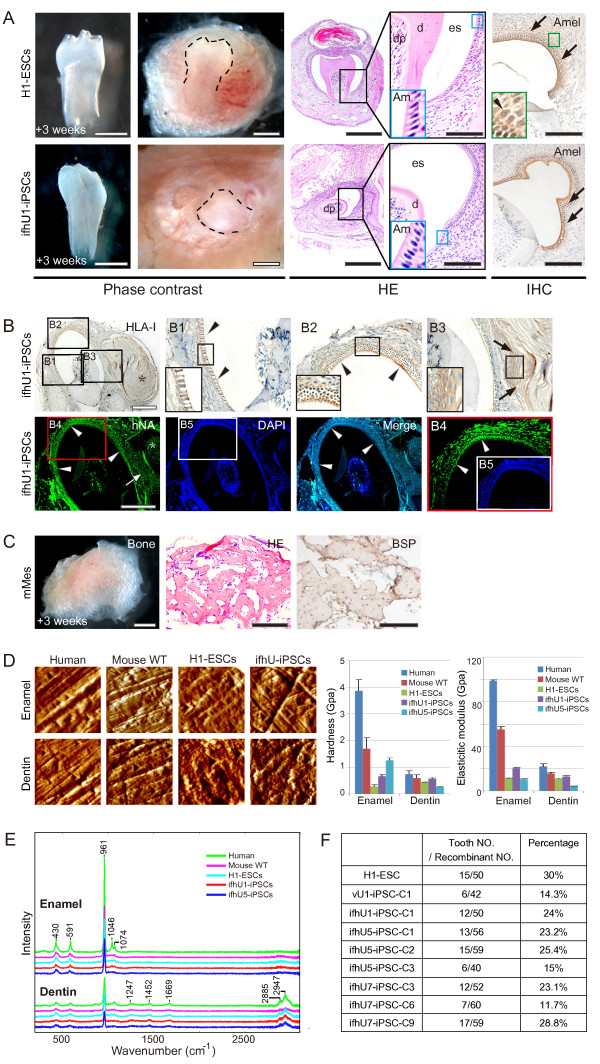
**Tooth**-**like structures formed from H1**-**ESC line and hiPSC lines in 3 weeks.** **(A)** Left two columns: tooth-like structures after and before isolated from the surrounding tissues from H1-ESC and ifhU1-iPSC groups (tooth is outlined by a dotted line); right three columns: HE and immunohistochemical staining (IHC) of relative tooth sections (H1-ESC: sagittal section; ifhU1-iPSC: cross section), showing the tooth-like structures containing dental pulp (dp), dentin (d), enamel space (es), and a layer of ameloblasts (Am, high magnification in the blue box) in both groups. Positive Amel (black arrows) expresses in these ameloblasts with the papillary layer ( black arrowheads at high magnification in the green box). Scale bar: the upper and bottom figures in the same column share the same scale bar as 500, 500, 400, 100, 100 μm from left to right orderly. **(B)** IHC (top, HLA-I in cytoplasm) and IF (bottom, hNA in nucleus) staining in cross sections of the ifhU1-iPSCs derived tooth: human iPSC derived cells and tissues including ameloblast layer (arrowheads), cyst epithelial cells (arrows) and epithelium-derived cyst (*). DAPI is shown in blue. Scale bars: 200 μm. **(C)** Image of a piece of bone detected from E14.5 mouse dental mesenchyme being transplanted under kidney capsule for 3 weeks with positive BSP expression. Scale bars: 1000, 100, 100 μm orderly. **(D)** Nano-indentation analyses, Left: representative images of fractured enamel and dentin surfaces in adult human tooth group (human), group of 3-week mouse tooth from E14.5 tooth germs (Mouse-WT), regenerative tooth groups from H1-ESCs and ifhU-iPSCs; right: hardness and elastic modulus of enamel and dentin in above groups (each group: n=3). **(E)** Raman spectroscopy analyses of enamel and dentin from human, mouse, hESCs, and ifhU-iPSCs showed great similarity for all groups on Raman peaks. **(F)** Efficiencies of tooth-like structures for H1-ESC line and 8 iPSC lines.

We then harvested D7 epithelial sheets and recombined them with the mouse dental mesenchyme before transplantation into mouse subrenal capsule (Additional file 1). After 3 weeks, we observed tooth-like structures with the fibrous cysts in the kidney (Figure 2A). We isolated individual tooth-like structures by removing them from the fibrous cysts and the surrounding bone (Figure 2A, left columns). We found that the tooth-like structures always appeared with the presence of fibrous cysts. The tooth-like structure contained dental pulp, dentin, enamel space, and enamel organ (Figure 2A, middle columns). The enamel organs have elongated ameloblasts with a ruffled border-like structure and papillary layer (Figure 2A, middle columns). We also observed the expression of Ameloblastin (Amel) located in the layer of ameloblasts and its papillary layer (Figure 2A). We confirmed the human origin of the epithelial component in cross sections of recombinant tooth prior to isolation by immunostainings with human specific antibodies against human leukocyte antigen-I (HLA-I) and human nucleus antigen (hNA) (Figure 2B). Both antibodies stained negatively in the dental pulp, cartilage, surrounding bone-like structures, which were developed from mouse dental mesenchyme (Figure 2B). As expected, both human specific antibodies stained positively for the ameloblasts (Figures 2B1 and 2B4), papillary layer besides (Figures 2B2 and 2B4), and squamous epithelial cells in the cyst (Figure 2B3). Furthermore, positive HLA-I staining was localized in the cytoplasm (Figures 2B1-2B3), while hNA was complementarily localized in the nucleus (Figures 2B4 and 2B5). As control, without recombination with hESCs or ifhU-iPSCs derived epithelial sheets, mouse dental mesenchymes transplanted under identical conditions formed bone-like structures instead (n=10/10), as confirmed by positive staining of bone sialoprotein (BSP) in the whole bone-like structure embedded with osteocytes (Figure 2C).

We then analyzed the hardness and elastic modulus of human adult teeth (human group), 3-week mouse teeth developed from tooth germs under kidney capsule (Mouse WT group), and the regenerative teeth from H1-ESCs and ifhU-iPSCs groups by Nano-indentation (Figure 2D). The harness and elastic modulus of dentin and enamel in above five groups showed similar properties (Figure 2D). In the case of enamel, the hardness and elastic modulus of H1-ESCs and two ifhU-iPSCs groups were lower than those of human and Mouse WT groups (Figure 2D, right graphs). Interestingly, the hardness of ifhU5-iPSCs group was around 4 times higher than that of H1-ESCs group (Figure 2D, right graphs). Impressively, the hardness of ifhU5-iPSCs group reached 1/3 by comparison with that of human group (Figure 2D, right graphs). We further analyzed the chemical composition of the teeth by Raman spectroscopy. We showed that they all have similar spectra with comparable intensity (Figure 2E). In the five groups examined, the enamel of the teeth are highly mineralized and the spectra showed almost the same spectrum as hydroxyapatite [Ca_5_(PO_4_)_3_OH, the primary mineral in teeth] (data not shown). These tooth-like structures also showed similar signs of protein with Raman peaks in dentin, including the hydroxyapatite peak compared to human teeth (Figure 2E). These results indicated that the regenerative teeth from both H1-ESCs and two ifhU-iPSCs groups have similar constituents with human and mouse teeth. We analyzed a total of 8 hiPSC lines from three urine donors (U1, 5, 7) for tooth regeneration and had a success rate of up to 30% (Figure 2F).

## Discussion

In this study, we demonstrate that epithelial sheets derived from ifhU-iPSCs can functionally substitute tooth germ epithelium to regenerate tooth-like structures when combined with mouse dental mesenchyme *in vivo*. Recently, two reports have showed that mouse iPSCs could be induced to differentiate into amelobastin-expressing dental epithelial cells and odontogenic mesenchymal cells through neural crest-like cells respectively [19, 20]. In contrast, our approach provides an ideal model for the regeneration of patient specific tooth in the future.

It is well known that adult dental stem cells have been successfully applied in tissue engineering research. It has proven to be possible to generate dentin pulp complexes, even whole teeth out of isolated cells [25]. However, some required and major issues need to be solved before these developing approaches being translated to the dental clinic. Probably the most important limiting factor is the absence of consistent sources of epithelial stem cells with odontogenic potential in the adult human individual. Periodontal ligament cells and postnatal oral mucosal epithelial cells are considered as the possible epithelial substitutes. Recently, human newborn skin keratinocyte progenitors were used to form tooth structures by recombination. However, FGF proteins are required for the formation of ameloblasts [26]. More recently, adult human gingival epithelial cells were reported to give rise to ameloblast-like cells in an bioengineered tooth when combined with mouse dental mesenchyme [27]. Although these approaches could lead to tooth formation, the sources of endogenous dental epithelial stem cells seem to be scarce. Thus, an appealing alternative would be to obtain the epithelial substitutes from autogenic iPSCs. Here, we succeeded in inducing intergration-free human urine iPSCs into the intact epithelial sheet, which developed into ameloblasts in a tooth-like structure. This positive experience indicates that human iPSCs could be applied on tooth regeneration.

Although our approach displayed iPSC contribution only to the epithelial substitutes, a dental engineering process generated from human cells could be easily designed. As the ectomesenchymal stem cells, such as human dental pulp stem cells can generate a complete dentin-pulp complex and peridontium. The hard enamel tissue constituting the remaining part of the tooth could be formed by human iPSC derived epithelial sheet described here. Therefore, success of our approach confirmed that autogenic iPSCs could be used as a source of new dental epithelium to be recombined with ectomesenchymal cells, thus creating a bioengineered tooth germ that can be cultured *in vitro* and transplanted to the jawbone of a recipient host to form a fully function tooth [10]. Of course, it remains more work for generating our iPSC derived epithelial sheet with odontogenic potential based on detailed analysis of molecular events associated epithelial-mesenchymal interactions, a theme recurrent throughout development [28–30]. On the other hand, future work should focus on the derivation of odontogenic cells and/or dental mesenchyme from ifhU-iPSCs which will be useful for the final dream of total regeneration of human tooth for clinical therapy.

## Conclusions

In conclusion, human iPSCs are capable of contribution on tooth generation by pre-differentiating into epithelial sheets and further responding to odontogenic signals from embryonic dental mesenchyme. In particular, these regenerative teeth contain enamel with ameloblast-like cells of human origin and possess physical properties found in the regular human tooth. Thus, human iPSCs could be a candidate source of seed cells on human tooth tissue-engineering for further drug screening or regenerative therapies.

## Methods

### Cell culture and epithelial differentiation

We used 9 distinct lines of pluripotent cells in this study. These included hESCs (WA01/H1; WiCell, USA). 8 hiPSC lines derived from urine cells of three donors (U1, 5, 7) were obtained from South Stem Cell Bank in China. Epithelial differentiation of hESCs and iPSCs was carried out with differentiation medium (DMEM/F12 [Gibco] supplemented with 1× N2 supplement, 1 μM RA and 25 ng/ml BMP4) when hESCs or iPSCs reach 70-80% confluence. Medium was replaced directly at D7 with defined keratinocyte serum-free medium and supplement (DSFM) to the confluent cell layer. In addition, differentiated cells at D7 could be detached with Dispase (BD, Cat. NO. 17105–041) and distributed on gelatin-coated plates at a split ratio of 1:3 with culture medium (DSFM) changing every other day till D42. Detailed methods and any associated references are available in an Additional file 2.

### Reconstitution of bioengineered tooth germs and subrenal capsule assays

The molar tooth germs were dissected from the mandibles of E14.5 mice and incubated in 0.75 mg/ml Dispase (BD) for 40 min at 37°C. The dental mesenchyme was separated from the dental epithelium. Meanwhile, hESCs or iPSCs derived epithelial cells at D7 were harvested as an intact epithelial sheet using 2 mg/ml Dispase (BD). A piece of dissected epithelial sheet was placed on the top of one mouse dental mesenchyme. The recombinant explants were cultured with Trowell-type system in DMEM containing 10% FBS for 1–2 days before being transplanted into the renal subcapsular layer of adult nude mice. Usually, 6–8 recombinant specimens were transplanted into per kidney. After 3 weeks, the host mice were sacrificed, and kidneys were dissected to obtain the calcified tissues.

### Immunohistochemistry

The calcified tissues were decalcified and sectioned in a 5 μm thickness. For immunohistochemistry, the primary antibodies used were anti-Ameloblastin (Amel; 1:100, Santa), anti-human leukocyte antigen I (HLA-I, 1:50, abcam) and anti-bone sialoprotein (BSP; 1:100, Millipore) polyclonal antibodies. For immunofluorescence, the primary antibodies used were anti-human nucleus antigen (hNA, 1:500, Millipore), anti-Cytokeratin 14 (K14; 1:100, Santa) and anti-p63 (p63; 1:100, Invitrogen) polyclonal antibodies.

### Real-time quantitative PCR

Total RNA was extracted with Trizol (Invitrogen). 2 μg of RNA was reverse transcribed using RT-PCR kit (Takara) and qPCR was performed using a Thermal Cycler Dice7™ Real Time System and SYBR Green Premix EX Taq™ (Takara).

### Western blot

The primary antibodies used were anti-Oct4 (Oct4 pAb; 1:1000), anti-Cytokeratin 18 (K18 pAb; 1:1000) and anti-p63 (p63 pAb; 1:1000) polyclonal antibodies. Anti-mouse or goat HRP was used to detect protein together with the Amersham ECL kit (GE Healthcare).

### Scanning electronic microscope (SEM) and transmission electron microscopy (TEM)

The differentiating cells at D7, D14 were prefixed with 2.5% glutaraldehyde for 24 h at 4°C, and postfixed with 1% osmium tetroxide for 2 h. For SEM, the samples were examined by scanning electron microscopy (PHILIPS, XL-30ESEM). For TEM, the samples were examined with transmission electron microscope (PHILIPS, TECNAI-10).

### Nano-indentation analyses

The teeth profiles were exposure after embedded with epoxy resins and opened packet buy piece for further examination. Nano indentation hardness calculation formula is: H = P max/A (h c). [H: hardness; P max: the largest indentation force; A (h c): projection area of the indentation].

### Raman spectroscopy assay

Spontaneous Raman spectra were acquired with a confocal Raman spectrometer (Labram HR800, Horiba JobinYvon) under 784.4 nm excitation laser through a 50x objective. The integration time was 10 s. The spectrometer was calibrated with the Raman line of silicon at 520.7 cm^-1^.

## Electronic supplementary material

Additional file 1: **Schematic representation of procedures for tooth generation using hESCs and hiPSCs.** The hESCs or hiPSCs differentiated into a piece of epithelial sheet, which was sliced into 1-mm^2^ squares and each then recombined with an E14.5 dental mesenchyme separated from dental epithelium of ICR mouse molar tooth germ. The recombinants were cultured *in vitro* for 1–2 days followed by HE staining for the developing structure and hNA antibody reaction for confirmation of human cell origin. Meanwhile, 6–8 cultured recombinants were transplanted beneath a subrenal capsule for 3 weeks for further calcified tooth formation. Scale bar: 300 μm. (PDF 736 KB)

Additional file 2: **Detailed methods.** (DOCX 23 KB)

## Abbreviations

ifhU-iPSCs: integration-free human urine induced pluripotent stem cells
E14.5: Embryonic day 14.5
hESC: human embryonic stem cell
DPSCs: Dental pulp stem cells
PDLSCs: Periodontal ligament stem cells
GSCs: Gum stem cells
iPSCs: induced pluripotent stem cells
miPSCs: mouse iPSCs
K14: cytokeratin-14
DSP: Dentin sialoprotein
hU: human urine cells
RA: Retinoic acid
BMP4: Bone morphogenetic protein 4
DSFM: Defined keratinocyte serum-free medium and supplement
SEM: Scanning electronic microscope
TEM: Transmission electron microscopy
Amel: Ameloblastin
HLA-I: Human leukocyte antigen-I
hNA: human nucleus antigen
BSP: Bone sialoprotein
DMEM: Dulbecco’s modified eagle’s medium
FBS: Fetal bovine serum
IF: Immunofluorescence
HE: Hematoxylin-Eosin staining
IHC: Immunohistochemical staining.

## References

[1] Brockes JP, Kumar A (2005). Appendage regeneration in adult vertebrates and implications for regenerative medicine. Science.

[2] Nishikawa S, Goldstein RA, Nierras CR (2008). The promise of human induced pluripotent stem cells for research and therapy. Nat Rev Mol Cell Biol.

[3] Takahashi K, Yamanaka S (2006). Induction of pluripotent stem cells from mouse embryonic and adult fibroblast cultures by defined factors. Cell.

[4] Chen YF, Tseng CY, Wang HW, Kuo HC, Yang VW, Lee OK (2012). Rapid generation of mature hepatocyte-like cells from human induced pluripotent stem cells by an efficient three-step protocol. Hepatology.

[5] Fujimoto Y, Abematsu M, Falk A, Tsujimura K, Sanosaka T, Juliandi B, Semi K, Namihira M, Komiya S, Smith A (2012). Treatment of a mouse model of spinal cord injury by transplantation of human induced pluripotent stem cell-derived long-term self-renewing neuroepithelial-like stem cells. Stem Cells.

[6] Hargus G, Cooper O, Deleidi M, Levy A, Lee K, Marlow E, Yow A, Soldner F, Hockemeyer D, Hallett PJ (2010). Differentiated Parkinson patient-derived induced pluripotent stem cells grow in the adult rodent brain and reduce motor asymmetry in Parkinsonian rats. Proc Natl Acad Sci USA.

[7] Wang Y, Zheng CG, Jiang Y, Zhang J, Chen J, Yao C, Zhao Q, Liu S, Chen K, Du J (2012). Genetic correction of beta-thalassemia patient-specific iPS cells and its use in improving hemoglobin production in irradiated SCID mice. Cell Res.

[8] Tucker A, Sharpe P (2004). The cutting-edge of mammalian development; how the embryo makes teeth. Nat Rev Genet.

[9] Nakao K, Morita R, Saji Y, Ishida K, Tomita Y, Ogawa M, Saitoh M, Tomooka Y, Tsuji T (2007). The development of a bioengineered organ germ method. Nat Methods.

[10] Ikeda E, Morita R, Nakao K, Ishida K, Nakamura T, Takano-Yamamoto T, Ogawa M, Mizuno M, Kasugai S, Tsuji T (2009). Fully functional bioengineered tooth replacement as an organ replacement therapy. Proc Natl Acad Sci USA.

[11] Chai Y, Jiang X, Ito Y, Bringas P, Han J, Rowitch DH, Soriano P, McMahon AP, Sucov HM (2000). Fate of the mammalian cranial neural crest during tooth and mandibular morphogenesis. Development.

[12] Pispa J, Thesleff I (2003). Mechanisms of ectodermal organogenesis. Dev Biol.

[13] Kollar EJ, Fisher C (1980). Tooth induction in chick epithelium: expression of quiescent genes for enamel synthesis. Science.

[14] Mina M, Kollar EJ (1987). The induction of odontogenesis in non-dental mesenchyme combined with early murine mandibular arch epithelium. Arch Oral Biol.

[15] Thesleff I, Tummers M: **Tooth organogenesis and regeneration.** In *StemBook*. Cambridge (MA): Harvard Stem Cell Institute; [Internet: http://www.stembook.org/] [Internet: ] 20614625

[16] Gronthos S, Mankani M, Brahim J, Robey PG, Shi S (2000). Postnatal human dental pulp stem cells (DPSCs) in vitro and in vivo. Proc Natl Acad Sci USA.

[17] Seo BM, Miura M, Gronthos S, Bartold PM, Batouli S, Brahim J, Young M, Robey PG, Wang CY, Shi S (2004). Investigation of multipotent postnatal stem cells from human periodontal ligament. Lancet.

[18] Mao JJ, Prockop DJ (2012). Stem cells in the face: tooth regeneration and beyond. Cell Stem Cell.

[19] Arakaki M, Ishikawa M, Nakamura T, Iwamoto T, Yamada A, Fukumoto E, Saito M, Otsu K, Harada H, Yamada Y (2012). Role of epithelial-stem cell interactions during dental cell differentiation. J Biol Chem.

[20] Otsu K, Kishigami R, Oikawa-Sasaki A, Fukumoto S, Yamada A, Fujiwara N, Ishizeki K, Harada H (2012). Differentiation of induced pluripotent stem cells into dental mesenchymal cells. Stem Cells Dev.

[21] Wen Y, Wang F, Zhang W, Li Y, Yu M, Nan X, Chen L, Yue W, Xu X, Pei X (2012). Application of induced pluripotent stem cells in generation of a tissue-engineered tooth-like structure. Tissue Eng Part A.

[22] Yu J, Hu K, Smuga-Otto K, Tian S, Stewart R, Slukvin II, Thomson JA (2009). Human induced pluripotent stem cells free of vector and transgene sequences. Science.

[23] Liao B, Bao X, Liu L, Feng S, Zovoilis A, Liu W, Xue Y, Cai J, Guo X, Qin B (2011). MicroRNA cluster 302–367 enhances somatic cell reprogramming by accelerating a mesenchymal-to-epithelial transition. J Biol Chem.

[24] Metallo CM, Ji L, De Pablo JJ, Palecek SP (2008). Retinoic acid and bone morphogenetic protein signaling synergize to efficiently direct epithelial differentiation of human embryonic stem cells. Stem Cells.

[25] Volponi AA, Pang Y, Sharpe PT (2010). Stem cell-based biological tooth repair and regeneration. Trends Cell Biol.

[26] Wang B, Li L, Du S, Liu C, Lin X, Chen Y, Zhang Y (2010). Induction of human keratinocytes into enamel-secreting ameloblasts. Dev Biol.

[27] Angelova Volponi A, Kawasaki M, Sharpe PT (2013). Adult Human Gingival Epithelial Cells as a Source for Whole-tooth Bioengineering. J Dent Res.

[28] Chen Y, Zhang Y, Jiang TX, Barlow AJ, St Amand TR, Hu Y, Heaney S, Francis-West P, Chuong CM, Maas R (2000). Conservation of early odontogenic signaling pathways in Aves. Proc Natl Acad Sci USA.

[29] Kangas AT, Evans AR, Thesleff I, Jernvall J (2004). Nonindependence of mammalian dental characters. Nature.

[30] Jernvall J, Thesleff I (2012). Tooth shape formation and tooth renewal: evolving with the same signals. Development.

