# Supplementary figures and images for: Generation of tooth-like structures from integration-free human urine induced pluripotent stem cells
Source: Cell Regen. 2013 Jul 30;2:6. doi: 10.1186/2045-9769-2-6 (PMC4230506; doi:10.1186/2045-9769-2-6)

# Additional file 1

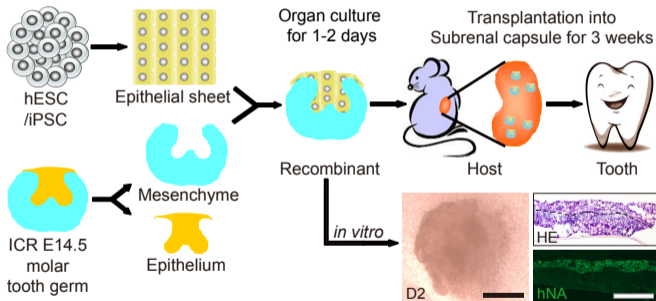

Supplement: Supplementary file 1 — Additional file 1: Schematic representation of procedures for tooth generation using hESCs and hiPSCs. The hESCs or hiPSCs differentiated into a piece of epithelial sheet, which was sliced into 1-mm2 squares and each then recombined with an E14.5 dental mesenchyme separated from dental epithelium of ICR mouse molar tooth germ. The recombinants were cultured in vitro for 1–2 days followed by HE staining for the developing structure and hNA antibody reaction for confirmation of human cell origin. Meanwhile, 6–8 cultured recombinants were transplanted beneath a subrenal capsule for 3 weeks for further calcified tooth formation. Scale bar: 300 μm. (PDF 736 KB) [file 13619_2013_15_MOESM1_ESM.pdf]
