# Supplementary material for: Generation of tooth-like structures from integration-free human urine induced pluripotent stem cells
Source: Cell Regen. 2013 Jul 30;2:6. doi: 10.1186/2045-9769-2-6 (PMC4230506; doi:10.1186/2045-9769-2-6)
Supplement: Supplementary file 2 — Additional file 2: Detailed methods. (DOCX 23 KB) [file 13619_2013_15_MOESM2_ESM.docx]

**Methods Section**

**Animals**

ICR mice and nude mice were purchased from Vital River Laboratories (Beijing, China). Mouse care and handling conformed to the NIH guidelines for animal research. All experimental protocols were approved by Guangzhou Institutes of Health Animal Care and Use Committee.

**Cell culture and epithelial differentiation**

All human samples were used following principles approved by the Guangzhou Institutes of Biomedicine and Health Ethical Committee. H1 hESC line used here was purchased from WiCell (WA01/H1; WiCell, USA). 8 hiPSC lines derived from urine cells of three donors (U1, 5, 7) were obtained from South Stem Cell Bank in China. 1 hiPSC line (vU1-iPSC-C1) was generated from NO.1 donor (U1) in a retrovirus system [[1](#_ENREF_1)]; the other 7 integration-free hiPSCs lines generated via transfection with oriP/EBNA episomal vectors. H1-hESCs and hiPSCs were maintained their pluripotency in mTesR1 medium (STEMCELL) on Matrigel-coated plates, and routinely passaged every 4-5 days using 0.5 mM EDTA.

Epithelial differentiation of hESCs and iPSCs was carried out as described previously [[2](#_ENREF_2)]. Briefly, it began the treatment with differentiation medium (DMEM/F12 [Gibco] supplemented with 1× N2 supplement [Invitrogen, Cat. NO. 17504-044], 1 μM all-trans retinoic acid [RA, Sigma, Cat. NO. R2625], and 25 ng/ml BMP4 [R&D Systems, Cat. NO. [314-BP-050](http://www.rndsystems.com/pdf/314-bp.pdf)]) when hESCs or iPSCs reach 70-80% confluence in 6-well plates. Medium was changed daily to 7 days and then replaced directly with defined keratinocyte serum-free medium and supplement (DSFM, Invitrogen, Cat. NO. 10744-019) to the confluent cell layer. DSFM was changed every other day for 3 weeks (D28). In addition, differentiated cells at D7 could be detached with Dispase (BD, Cat. NO. 17105-041) and distributed on gelatin-coated plates at a split ratio of 1:3 with culture medium (DSFM) changing every other day till D42.

**Reconstitution of bioengineered tooth germs**

The molar tooth germs were dissected from the mandibles of E14.5 mice and incubated in 0.75 mg/ml Dispase (BD) for 40 min at 37℃. The tooth germs were washed in DMEM with 10% FBS, and the dental mesenchyme was separated from the dental epithelium. Meanwhile, hESCs or iPSCs derived epithelial cells at D7 were harvested as an intact epithelial sheet using 2 mg/ml Dispase (BD). After dissecting into pieces as a similar size of the mouse E14.5 dental mesenchyme, a piece of epithelial sheet was placed on the top of one mouse dental mesenchyme. The recombinant explants were cultured with Trowell-type system in DMEM containing 10% FBS for 1-2 days.

**Subrenal capsule assays**

The recombinant tooth germs and the mouse dental mesenchyme without recombination were transplanted into the renal subcapsular layer of adult nude mice respectively. For well development, 6-8 specimens were usually transplanted into per kidney. All surgical procedures were performed under anesthesia administered intra-peritoneally. No immunosuppressive medication was used. After 3 weeks, the host mice were sacrificed, and kidneys were dissected to obtain the calcified tissues.

**Specimen preparation and immunohistochemistry**

The differentiated cells and in vitro culture tooth germs were fixed in 4% paraformaldehyde in PBS. The calcified tissues the tissues were decalcified in 10% EDTA (pH 7.4) for 4 weeks at room temperature and sectioned in a 5 μm thickness and then stained with H&E. For immunohistochemistry, the primary antibodies used were anti-Ameloblastin (Amel; 1:100, Santa, Cat. NO. sc-50534), anti-human leukocyte antigen I (HLA-I, 1:50, abcam, Cat. NO. ab70328) and anti-bone sialoprotein (BSP; 1:100, Millipore, Cat. NO. AB1854) polyclonal antibodies. For immunofluorescence, the primary antibodies used were anti-human nucleus antigen (hNA, 1:500, Millipore, Cat. NO. 1969098), anti-Cytokeratin 14 (K14; 1:100, Santa, sc-17104) and anti-p63 (p63; 1:100, Invitrogen, 1117634A) polyclonal antibodies. The sections were observed using an Axio Scope A1 (Carl Zeiss, Jena, Germany) with an AxioCAM MRc5 (Zeiss) and processed with AxioVision software (Zeiss).

**Real-time quantitative PCR**

Total RNA was extracted with Trizol (Invitrogen). 2 µg of RNA was reverse transcribed using RT-PCR kit (Takara) and qPCR was performed using a Thermal Cycler Dice^TM^ Real Time System and SYBR Green Premix EX Taq^TM^ (Takara). GAPDH was used for qPCR normalization and all items were measured in triplicate. Primer sequences are as follows:

*Oct4* forward “GCCGTGAAGCTGGAGAAGGA”

reverse “GAACCACACTCGGACCACAT”

*p63* forward “GGAAAACAATGCCCAGACTC”

reverse “GTGGAATACGTCCAGGTGG”

*CD29* forward “ATGCCTACTTCTGCACGATG”

reverse “TCCTTTGCTACGGTTGGTTA”

*K14* forward “GTGGTGATGGGCTTCTGG”

reverse “CCATTGATGTCGGCTTCC”

*K18* forward “AAGAACCACGAAGAGGAAGTAAA”

reverse “CTGTCCAAGGCATCACCAAG”

*K19* forward “GCGACTACAGCCACTACTACACGA”

reverse “TCTTCCAAGGCAGCTTTCATG”

*GAPDH* forward “AAGGTGAAGGTCGGAGTCAAC”

reverse “GGGGTCATTGATGGCAACAATA”

**Western blot**

A 12% acrylamide gel was run at 120 Volt for 1 hour. Transfer was performed using nitrocellulose for 1 hour at 100 Volt. The primary antibodies used were anti-Oct4 (Oct4 pAb; 1:1000, Cell Signaling, Cat. NO. 2750), anti-Cytokeratin 18 (K18 pAb; 1:1000, Santa, sc-51583) and anti-p63 (p63 pAb; 1:1000, Invitrogen) polyclonal antibodies. Anti-mouse or goat HRP was used to detect protein together with the Amersham ECL kit (GE Healthcare).

**Scanning electronic microscope (SEM) and transmission electron microscopy (TEM)**

The differentiating cells at D7, D14 were prefixed with 2.5% glutaraldehyde for 24 h at 4 °C, and postfixed with 1% osmium tetroxide for 2 h. For SEM, the samples were then dehydrated in a graded series of ethanol, followed by critical point drying using a HCP-2 apparatus (Hitachi, Tokyo, Japan) with CO_2_ as the transitional fluid. The specimens, which were mounted on stubs, were coated with platinum and examined by scanning electron microscopy (PHILIPS, XL-30ESEM). For TEM, the samples were dehydrated in graded ethyl alcohols and embedded with Epon812. An ultrathin section was cut with an Ultracut Eultramicrotome, stained with uranyl acetate and lead citrate, and then examined with transmission electron microscope (PHILIPS, TECNAI-10).

**Nano-indentation analyses**

The teeth profiles of hESCs/iPSCs regenerative teeth and 3-week mouse teeth or the human adult teeth were exposure after embedded with epoxy resins and opened packet buy piece. Mechanically polished up to 2000 grit, the sample were attached to the nanometer indentation special sample stage of the nano indenter (HysitronInc company Tribo Indenter ) with the diamond triangular pyramid shaped head, 142.35°, curvature radius 50 nm. Load set to 1 mN, 10 seconds (s) loading, maintain 10 s, then 10 s unloading. Each sample was tested for 3-4 points. Nano indentation hardness calculation formula is: H = P max/A (h c). [H: hardness; P max: the largest indentation force; A (h c): projection area of the indentation]

**Raman spectroscopy assay**

Spontaneous Raman spectra were acquired with a confocal Raman spectrometer (Labram HR800, Horiba JobinYvon) under 784.4 nm excitation laser through a 50x objective. The integration time was 10 s and the measurement was repeated for twice. The spectrometer was calibrated with the Raman line of silicon at 520.7 cm^-1^. Baseline correction was performed in the software LabSpec.

**References**

[1] Zhou T, Benda C, Duzinger S*, et al.* Generation of induced pluripotent stem cells from urine. Journal of the American Society of Nephrology : JASN, 2011, 22: 1221-1228

[2] Metallo CM, Ji L, de Pablo JJ*, et al.* Retinoic acid and bone morphogenetic protein signaling synergize to efficiently direct epithelial differentiation of human embryonic stem cells. Stem Cells, 2008, 26: 372-380
